# Auditory neural entrainment: frequency-specific functional roles and emerging applications in neurological and psychiatric disorders

**DOI:** 10.3389/fnhum.2026.1934763

**Published:** 2026-09-02

**Authors:** Hua Yang, Yan Li, Ziyi Chen, Jing Lu

**Affiliations:** 1 Department of Composition, Sichuan Conservatory of Music, Chengdu, Sichuan, China; 2 Key Laboratory for Music Digital Intelligence of Sichuan, Sichuan Conservatory of Music, Chengdu, Sichuan, China; 3 The Clinical Hospital of Chengdu Brain Science Institute, MOE Key Lab for Neuroinformation, University of Electronic Science and Technology of China, Chengdu, Sichuan, China; 4 School of Life Science and Technology, Center for Information in Medicine, University of Electronic Science and Technology of China, Chengdu, Sichuan, China

**Keywords:** auditory entrainment, cross-frequency coupling, gamma stimulation, neural oscillations, neurological and psychiatric disorders

## Abstract

Among sensory inputs, auditory information plays a significant role. Neural entrainment may establish a bridge for the brain’s perception of rhythmic sound signals. It refers to the synchronization of ongoing neuronal activity with internal and external rhythmic stimuli, demonstrating the brain’s plasticity and adaptive dynamics. Across canonical EEG bands, delta activity is associated with temporal prediction and hierarchical auditory processing over longer time scales; theta activity with speech parsing, audiovisual integration, and memory-related timing; alpha activity with cross-modal information processing; beta activity with predictive timing and auditory-motor coordination; and gamma-range responses with fine temporal encoding and steady-state activity. These associations overlap rather than represent one-to-one functional mappings. This mini review included studies based on speech, music, pure tones, and other natural or artificial auditory stimuli. We explored the functional roles of these oscillatory activities and their interactions. Finally, we prudently reviewed the emerging applications of auditory neural entrainment for brain diseases. These explorations have yielded some exciting results, but the evidence remains in its early stages and shows heterogeneity. It is also limited by small sample sizes and short study periods. Moreover, many music- and rhythm-based interventions have shown symptom improvement, but most provide only indirect evidence. Therefore, we emphasize that future studies should more deeply explore the relationship between neural entrainment and proven therapeutic benefits, while also attending to safety, individual differences, and the adequacy of control groups in sham interventions.

## Introduction and brief historical perspective

Rhythmic auditory input enables neural responses to match the temporal structure of the sound, thereby supporting prediction, segmentation, attention, memory, and sensory-motor coordination. The modern entrainment framework stems from multiple interrelated research directions. Speech research shows that low-frequency cortical activity tracks the temporal structure across various time scales, while auditory temporal sequence research links EEG features to expectations and beat-based predictions (Giraud and Poeppel, 2012; Stefanics et al., 2010; Fujioka et al., 2012). Subsequently, studies on rhythmic stimulation emphasized that externally driven phase alignment may alter perception and also raised questions about how to distinguish endogenous oscillatory synchronization from repetitive-induced responses. These studies are of great significance for speech and music perception and have driven research on applying frequency-specific sensory stimuli to the nervous system and on the treatment of mental disorders, such as recent explorations of using 40 Hz sensory stimulation to intervene in cognitive aging-related diseases. Thus, precise use of terminology and evidence grading becomes particularly important, as they facilitate the distinction among physiological stimulus responses, activation of specific neural targets, and clinically significant therapeutic effects.

## Scope and literature-selection approach

For this review, auditory neural entrainment is defined as the externally driven alignment or adjustment of ongoing neural oscillatory activity to the temporal structure of an auditory stimulus. Strong evidence should extend beyond a spectral peak or a phase-locked response at the stimulation frequency. It should, where possible, demonstrate properties expected of an endogenous oscillator, such as phase-dependent modulation, frequency selectivity, persistence after stimulation, or effects that the linear superposition of repeated evoked responses cannot explain. Phase resetting may contribute to entrainment, whereas auditory steady-state responses (ASSR) and neural tracking are treated as measurable stimulus-following or target-engagement responses that, by themselves, do not prove entrainment of an endogenous oscillation. Cross-frequency coupling describes coordination among frequencies rather than entrainment per se. Auditory-motor synchronization, behavioral rhythm training, and general music therapy are considered distinct phenomena and are classified as indirect mechanistic evidence unless neural entrainment was both manipulated and measured.

PubMed was searched as the primary database, with supplementary searches in Web of Science and Google Scholar; the search was updated through 1 August 2026. Representative combinations included: auditory, acoustic, speech, music, rhythm, neural entrainment, phase synchronization, phase locking, auditory steady-state response, neural tracking, delta, theta, alpha, beta, gamma, cross-frequency coupling. Disorder-specific searches combined these terms with Alzheimer’s disease, ADHD, attention-deficit/hyperactivity disorder, autism, ASD, depression, or major depressive disorder. English-language, peer-reviewed studies were eligible. Publications from the previous 15 years were prioritized, while older seminal studies were retained when necessary. Animal mechanistic studies, human experimental studies, cross-sectional physiological studies, feasibility studies, pilot trials, and randomized controlled trials were considered but were not assigned equivalent evidential weight. Reviews were used for contextual synthesis and to identify primary studies; preprints and other unpublished material were excluded. Search results were screened for direct relevance to auditory/rhythmic stimulation, oscillatory measurements, or the target disorders. Representative mechanistic studies were selected to illustrate established associations and methodological controversies rather than to provide exhaustive coverage.

Table 1 was restricted to selected peer-reviewed human studies that either (i) delivered frequency- or rhythm-specific auditory stimulation and measured a neural response, (ii) reported clinically relevant outcomes from frequency-targeted stimulation, or (iii) provided clinically relevant rhythm/music evidence that was explicitly labeled indirect. “Physiological target engagement” required a frequency- or rhythm-specific intervention plus an objective neural measure related to the proposed target; “preliminary clinical signal” required a clinically relevant outcome but did not imply definitive efficacy; and “indirect evidence” denoted rhythm/music interventions without direct verification of neural entrainment.

**Table 1:** Selected peer-reviewed human studies relevant to the translation of auditory neural entrainment, stratified by evidence type.

Evidence type	Study	Population/sample	Design/control	Modality and parameters	Dose	Neural target engagement	Clinical outcome and principal result	Adverse events (AEs)	Major limitation
Direct, multisensory; preliminary clinical signal	Cimenser et al. (2021)	Mild-to-moderate AD; *n* = 22 (14 active, 8 sham)	Completer/subgroup analysis from an ongoing Phase I/II randomized, single-blind, sham-controlled trial; sham participants received high-frequency audiovisual stimulation not expected to evoke a steady-state response at the stimulation frequency.	40 Hz auditory + visual stimulation; sham designed not to evoke a 40 Hz response	1 h/day for 6 months	In-clinic EEG was used to configure a 40 Hz steady-state response, but quantitative participant-level target-engagement results were not presented in this report.	In the actigraphy subset (7 active, 6 sham), nighttime activity improved relative to sham; ADCS-ADL showed an exploratory favorable signal. Not definitive efficacy.	Potentially device-related AEs: 8/19 active, 4/12 sham; one severe dementia exacerbation in active group; one discontinuation per group; adherence high	Small, unequal groups; actigraphy rather than polysomnography.
Direct, multisensory; physiological target engagement and feasibility evidence.	He et al. (2021)	Prodromal AD, defined as MCI due to AD with positive CSF biomarkers; *n* = 10, randomized to two delayed-start groups of five participants each.	Randomized delayed-start feasibility study: 8 weeks of stimulation versus 4 weeks of no stimulation followed by 4 weeks of stimulation; no sham or placebo condition.	Combined 40 Hz visual and auditory flicker via goggles/headphones; individual tolerable intensity; auditory carrier/SPL incompletely reported	1 h/day at home for 4 or 8 weeks.	All enrolled participants showed a 40 Hz EEG response (mean 49/64 channels); entrainment at screening was required for enrollment; no increase in entrained channels with prolonged exposure.	Mean adherence 95.5%; PCC-precuneus connectivity increased after 8 weeks; core CSF Aβ/tau biomarkers unchanged; exploratory immune-factor changes; no clinical efficacy demonstrated.	No severe treatment-related AEs; mild possible events included dizziness, tinnitus, headache, and worsened hearing; screening intolerance/tinnitus and later headache-related discontinuation reported	Small sample; short duration; delayed-start design does not control expectancy; multiple exploratory endpoints; no evidence of clinical efficacy
Behavioral proof-of-concept rhythm training; indirect mechanistic evidence without neural verification.	Jamey et al. (2025)	Children age 7–13 with parent-reported ADHD; 31 enrolled, 27 completed, 26 analyzed (13/arm; 3 female, 1 non-binary)	Assessor-blinded randomized active-control study conducted remotely at home	Tablet auditory-motor rhythm game: finger taps synchronized to instrumental musical beats and audiovisual targets; active control used music/tapping without beat synchronization	Target 30 min/day, 5 days/week for 2 weeks; mean reported training 267 vs. 321.8 min; active gameplay time differed between groups	Not measured	Beat Tracking Index and tapping to music improved; metronome tapping and rhythm perception did not show clear group effects. Exploratory executive-function response-speed signal; no ADHD symptom/functional outcome.	Adverse events not systematically assessed; four discontinued and one completer excluded for invalid training data.	Small sample; diagnosis based on parental report; sex imbalance; brief intervention/no follow-up; missing data and unequal gameplay time; no verified double-blind; no neural or ADHD clinical-symptom measure
Cross-sectional physiological comparison; not an intervention	Liu et al. (2023)	Drug-naïve patients with first-episode major depressive disorder, *n* = 28, and healthy controls, *n* = 30; age 20–50 years, with normal hearing. All patients had HAMD-17 scores ≥14.	Cross-sectional case–control ASSR study.	Binaural 1-ms white-noise clicks at 40 and 60 Hz, 45 dB, 100% modulation depth; randomized order	3 sessions × 56 trials; 3-s stimulation/trial; 1.5-s inter-trial interval	ERSP and ITC at 40/60 Hz plus early theta ITC; ASSR power did not differ significantly between groups.-	Right-hemisphere 40 Hz ITC was lower in FEMD; 60-Hz ASSR-ITC showed no group difference; theta ITC during 60-Hz stimulation was reduced. Combined exploratory classifier AUC 0.868; severity associations were trend-level; no treatment outcome.	Not systematically assessed or reported;	Small cross-sectional sample; no causal/treatment inference; all patients at least moderately symptomatic; severity correlations trend-level; classifier lacked independent validation; cognition not assessed
Acute auditory frequency-sweep study with physiological spectral-response measurement; not a therapeutic intervention.	Shi et al. (2025)	Unipolar depression in remission; *n* = 141 (56 cognitive-impaired, 85 non-impaired)	Cross-sectional acute frequency sweep with between-subgroup comparison; no healthy/sham control	Auditory gamma-frequency entrainment sounds via earphones at 33–48 Hz; acoustic carrier, waveform, modulation method, SPL, and presentation order incompletely reported	20 s/frequency; 320 s stimulation plus 30-s rest before/after; about 7 min total	EEG power spectral density across canonical bands; no phase-locking or direct measure of endogenous entrainment	Gamma power did not differ between subgroups. The cognitively impaired subgroup showed lower alpha power during 37-Hz stimulation across multiple electrodes and at T4 during 48 Hz; small cognitive correlations; peak response frequency varied among participants. No clinical improvement tested.	Not systematically reported	Cross-sectional; no sham/healthy control or pre-post clinical comparison; spectral power does not establish entrainment; multiple correlation analyses; incomplete acoustic reporting; no longitudinal efficacy/safety; industry funding/involvement.
Indirect general music-therapy evidence; clinical behavioral outcomes without neural entrainment measurement.	Zhou et al. (2025)	Children with ASD; eligibility 30–80 months, enrolled range 31–80 months; *n* = 29 (15 music therapy, 14 standard care)	Assessor-masked parallel-group RCT; both groups received standard care; intervention group received additional music therapy.	Therapist-led group music therapy using Chinese songs, Orff instruments, singing, rhythmic movement, turn-taking, language practice, and social interaction; no fixed stimulation frequency	30 min, 3 times/week for 12 weeks; groups of 3–5; ~18 h scheduled treatment	Not measured	Greater improvement in SRS-2 social communication/total, ATEC speech-language-communication and sociability, and GDS personal-social DQ. ATEC total and GDS language/total did not show significant between-group improvement.	Not systematically reported	Small single-center sample; broad age/developmental range; no contact/time-matched active control; caregiver expectancy and multiple outcomes; no long-term follow-up; no neural measure; music/rhythm effects cannot be isolated from social-skills practice.
Frequency-targeted auditory stimulation embedded within adaptive cognitive training; preliminary controlled clinical signal without direct verification of gamma-band neural target engagement.	Wang et al. (2026)	Unipolar depression, age 12–60; 141 phenotyped; 56 cognitive-impaired participants allocated 1:1 at baseline. Final analytic sample stated as *n* = 46 (26 GE, 20 control), whereas flow diagram shows 26/22 at 8 weeks.	Reported randomized study; both groups received standard antidepressants; GE group additionally received n-back training + 40 Hz auditory stimulation; no matched sham/cognitive-training control.	20-min adaptive audiovisual n-back task; 40 Hz auditory component = 1-ms rectangular pulse every 25 ms through in-ear headphones; visual component was not 40 Hz flicker	~20 min/session, ≥5 days/week. The methods describe a 28-day treatment, but outcomes are reported at 4 and 8 weeks; the cumulative exposure through week 8 is unclear.	Direct 40 Hz engagement not measured. Resting microstate analysis used 2-20-Hz-filtered EEG and assessed GFP, duration, occurrence, coverage, and transitions.	MCCB total score improved more in GE at 4 and 8 weeks; selected domains differed at 8 weeks. Microstate measures showed temporal main effects without a clear intervention-specific group × time effect. Cognitive change was not associated with mood/anxiety change.	Safety and treatment-emergent adverse events were not systematically reported.	Inconsistent sample flow and treatment duration; attrition/LOCF; no sham or matched cognitive-training control; baseline HAMD imbalance; multicomponent intervention; broad age range; incomplete randomization/blinding details; microstates do not verify gamma entrainment; company-developed app/authors affiliated.
Frequency-targeted auditory–vibrotactile open-label pilot; preliminary clinical and resting-EEG signal without direct verification of neural target engagement.	Mosabbir et al. (2022)	MDD during a major depressive episode; 20 enrolled, 19 analysed; age 18–70; 11 female; 13/19 on stable psychiatric medication	Single-group open-label pre-post pilot; no randomized, sham, placebo, or active control; EEG analysis of same cohort as prior clinical report	Instrumental music with 30-70-Hz low-frequency components emphasizing 40 Hz plus 10-15-Hz binaural detunement; stereo auditory + lower-back vibrotactile stimulation; sound/vibration intensity not standardized	30 min/session, 5 days/week for 5 weeks; ~12.5 h scheduled exposure	Resting EEG power before/after treatment; no EEG during stimulation and no stimulus-locked 40 Hz phase/power measure	MADRS decreased from 26.84 to 18.36; 7/19 met ≥50% response. Eyes-open occipital alpha increased across full sample. Prefrontal gamma did not increase significantly across all participants, but change was greater in responders vs. nonresponders in an exploratory subgroup analysis.	Not systematically reported	Small open-label sample; no control or sham condition; no direct stimulus-locked target-engagement measure; inability to isolate the effects of 40 Hz, auditory stimulation, vibration, or music; concomitant stable psychiatric medication in most participants; non-standardized acoustic and vibration intensity.

Direct target engagement denotes frequency- or rhythm-specific stimulation with a neural measure. Indirect evidence refers to behavioral rhythm training or music-based interventions without neural verification.

AD, Alzheimer’s disease; ADCS-ADL, Alzheimer’s Disease Cooperative Study–Activities of Daily Living; ADHD, attention-deficit/hyperactivity disorder; AE, adverse event; ASSR, auditory steady-state response; ASD, autism spectrum disorder; ATEC, Autism Treatment Evaluation Checklist; AUC, area under the curve; Aβ, amyloid-beta; CSF, cerebrospinal fluid; DQ, developmental quotient; EEG, electroencephalography; ERSP, event-related spectral perturbation; FEMD, first-episode major depressive disorder; GDS, Gesell Developmental Schedules; GE, gamma entrainment; GFP, global field power; HAMD-17, 17-item Hamilton Depression Rating Scale; ITC, inter-trial phase coherence; LOCF, last observation carried forward; MADRS, Montgomery–Åsberg Depression Rating Scale; MCCB, MATRICS Consensus Cognitive Battery; MCI, mild cognitive impairment; MDD, major depressive disorder; MEG, magnetoencephalography; PCC, posterior cingulate cortex; RCT, randomized controlled trial; SPL, sound pressure level; SRS-2, Social Responsiveness Scale, Second Edition.

## Auditory neural entrainment and different EEG bands

Canonical frequency boundaries vary across studies, and the same frequency can reflect different physiological processes depending on the stimulus, cortical generator, behavioral context, recording modality, and analysis method. The following band-based organization should therefore be read as a heuristic summary of commonly observed associations, not as a set of one-to-one mappings between frequencies and functions.

### Delta EEG bands

Delta-wave activity is most commonly associated with temporal prediction and hierarchical auditory processing. Specifically, delta activity participates in the temporal prediction of auditory stimuli through rhythmic coupling (Etard and Reichenbach, 2019; Ding and Simon, 2012; Chang et al., 2019), responses to temporal deviations (Arnal et al., 2015), and temporal prediction encoding (Herrmann et al., 2016), and is related to the sensitivity of temporal prediction (ten Oever et al., 2017; Herbst and Obleser, 2019). Additionally, delta-wave synchronization is believed to be involved in the hierarchical processing of speech signals (Giraud and Poeppel, 2012; Keitel et al., 2018; Scott, 2019). This includes the more likely extraction of contextual information from acoustic signals over a longer timescale (Park et al., 2015), and greater participation in higher-level computational functions compared to theta synchronization (Molinaro and Lizarazu, 2018).

### Theta EEG bands

External rhythmic auditory stimulation can drive theta wave synchronization, thereby facilitating speech parsing and perceptual integration. The syllable rhythm of natural language is usually consistent with the theta frequency range. The auditory cortex appears to achieve temporal segmentation and information integration of continuous speech by tracking the theta waves of the speech envelope. However, theta-band activity does not specifically refer to auditory processing; it more broadly reflects attention sampling and memory processes. Auditory–visual speech can achieve synchronization of theta-wave activity across auditory, visual, multisensory, and memory-related networks (Michon et al., 2022; Biau et al., 2025; Hanslmayr et al., 2019). For example, studies have shown that visual stimulation at theta frequency can improve auditory working memory performance (Albouy et al., 2022), while synchronous auditory–visual stimulation can enhance memory performance (Clouter et al., 2017; Wang et al., 2018). Therefore, the theta-wave response may reflect the combined effects of mechanisms such as sensory tracking, phase resetting, memory coordination, and endogenous oscillation alignment.

### Alpha EEG bands

Traditionally, alpha-wave activity has been believed to be directly involved in visual processing (Jensen et al., 2014). The latest research shows that it can also be regulated by rhythmic auditory stimulation. Studies have demonstrated that both visual and auditory stimuli can lead to phase resetting of neural oscillations across sensory modalities (Bauer et al., 2020), a phenomenon known as cross-modal synchronization. Rhythmic sensory stimuli, including visual, auditory, and combined visual–auditory stimuli (Ronconi and Melcher, 2017), have been shown to trigger alpha-wave neural synchronization (de Graaf et al., 2013; Spaak et al., 2014; Ronconi et al., 2016; Ronconi et al., 2018).

### Beta EEG bands

Beta-wave activity is mainly involved in sensory prediction and auditory-motor coordination. In rhythmic sequences, beta power decreases after the event and rises before the anticipation, reflecting predictive time judgment rather than simple stimulus following (Fujioka et al., 2012; Cirelli et al., 2014). Beta oscillations participate not only in time prediction but also in predicting auditory content (Chang et al., 2016). For example, beta activity before the target can encode the predictability of the acoustic envelope and regulate time perception (Leske et al., 2025). Growing evidence suggests that beta oscillations are involved in auditory-motor interactions and sensory-motor coordination. In music and rhythm perception, beta oscillations may support the intrinsic maintenance of rhythmic structure and promote coordination between audition and movement (Fujioka et al., 2012; Cirelli et al., 2014; Morillon and Baillet, 2017). Overall, beta-wave synchronization appears to serve as a predictive mechanism, linking auditory perception to the motor and timing systems. However, changes in beta-wave power do not specifically indicate the predictive function and should be interpreted in conjunction with the task, sound-source localization, and behavioral measurements.

### Gamma EEG bands

Gamma-range responses are associated with fine temporal sampling, local sensory encoding, attention, and memory (Sahu et al., 2024; Teng et al., 2017; Crasta et al., 2018). Auditory gamma activity includes transient evoked responses and ASSRs, particularly around 40 Hz. These responses provide useful measures of neural synchrony and target engagement. However, a 40 Hz spectral response can arise from repeated evoked activity or linear stimulus following, and should not automatically be labeled as endogenous entrainment.

In recent studies, 40 Hz gamma-band stimulation has attracted considerable interest (Iaccarino et al., 2016; Adaikkan et al., 2019; Chan et al., 2021). Initial 40 Hz stimulation studies were conducted primarily in murine models. In one study (Martorell et al., 2019), 5XFAD mice (transgenic mice carrying five familial Alzheimer’s disease mutations and developing significant amyloid plaque loads) received auditory stimulation at various frequencies. The results indicated that 40 Hz auditory stimulation significantly reduced β-amyloid plaque load. Notably, combined 40 Hz audiovisual stimulation produced a more pronounced reduction in β-amyloid levels across the neocortex of 5XFAD mice than auditory or visual stimulation alone. These animal findings provide mechanistic evidence but cannot be generalized directly to human AD. In humans, 40 Hz auditory or audiovisual stimulation can evoke measurable steady-state responses and be delivered repeatedly, establishing feasibility and physiological target engagement. Whether these responses produce durable cognitive or functional benefit remains a separate clinical question.

### The relationships among different EEG bands

Auditory processing also depends on coordination across time scales. Delta and theta activity can provide temporal reference frames for continuous speech and music, beta activity can contribute to prediction and motor engagement, and gamma activity can support local encoding within slower cycles (Doelling et al., 2014; Doelling and Poeppel, 2015; Zalta et al., 2024; Samiee et al., 2022; Senkowski and Engel, 2024; Lizarazu et al., 2019; Lizarazu et al., 2023). Delta-beta coupling has been associated with predictive timing, pitch processing, groove, and multisensory temporal expectations (Chang et al., 2019; Arnal et al., 2015; Zalta et al., 2024; Samiee et al., 2022; Senkowski and Engel, 2024). Theta-gamma phase-amplitude relationships have been linked to speech-rate adaptation and language proficiency (Lizarazu et al., 2019; Lizarazu et al., 2023). These associations probably support a hierarchical account in which slow activity organizes temporal windows and faster activity represents local information.

Cross-frequency-coupling measures also require methodological caution. Apparent phase-amplitude coupling can arise from non-sinusoidal waveform shapes, harmonics, filtering choices, spectral leakage, or common stimulus-evoked transients rather than from a physiological interaction between distinct oscillators (Jensen et al., 2016; Lozano-Soldevilla et al., 2016). Credible inference therefore requires inspection of unfiltered signals, control for waveform asymmetry and harmonics, appropriate surrogate analyses, and replication across analytic approaches.

## Translational evidence and clinical applications

Since auditory neural entrainment can synchronize neural oscillations with external rhythmic stimuli, it has been increasingly investigated as an intervention for neurological and psychiatric diseases characterized by abnormal neural oscillations. Among these diseases, Alzheimer’s disease, attention-deficit hyperactivity disorder, autism spectrum disorder, and depression have drawn special attention. These conditions pose substantial challenges in the global public health domain. Therefore, we incorporated relevant literature on the application of auditory neural entrainment or auditory stimulation to these diseases.

### Applications in Alzheimer’s disease

AD is a neurodegenerative disease associated with aberrant neural oscillations. Research indicates that high-gamma synchrony declines in the elderly (Goossens et al., 2016) and that individuals with AD display aberrant gamma activity (Tichko et al., 2022).

Preclinical studies provide the strongest mechanistic support for gamma-frequency sensory stimulation in AD. In transgenic mouse models, auditory and multisensory 40 Hz stimulation has been associated with reduced amyloid-related pathology and altered microglial and network activity (Iaccarino et al., 2016; Adaikkan et al., 2019; Martorell et al., 2019). These findings establish biological plausibility in specific models, but differences in pathology, brain scale, stimulation exposure, and disease stage limit direct translation to human AD.

Human studies have primarily evaluated 40 Hz audiovisual rather than auditory-only stimulation. Feasibility work in amnestic mild cognitive impairment demonstrated measurable 40 Hz responses, acceptable adherence, and exploratory changes in connectivity or immune markers, without clear changes in core AD biomarkers (He et al., 2021). Small pilot studies in mild AD have reported target engagement and signals in sleep, daily functioning, structural measures, or cognition (Chan et al., 2022; Da et al., 2024; Cimenser et al., 2021). These outcomes should be described as feasibility findings or preliminary clinical signals, not established efficacy, because samples were small, endpoints were multiple, follow-up was limited, and many studies were not powered for clinical outcomes. A measurable 40 Hz response demonstrates physiological engagement; it does not establish disease modification.

Recently, in addition to using pure tones as stimuli, some have proposed an intervention that combines music with gamma-frequency stimulation (Tichko et al., 2022). One potential approach for AD combines gamma visual stimulation with musical elements. However, optimal methods for combining music with gamma auditory stimulation remain to be determined. Additionally, researchers have introduced an auditory stimulus termed “gamma music,” which merges a 40 Hz acoustic signal with musical components. This gamma music elicits a robust 40 Hz ASSR (Yokota et al., 2024) and is reported to be better tolerated than pure 40 Hz auditory stimulation. The above research has found some positive results. However, the efficacy of using a fixed 40 Hz frequency intervention in the population may be limited. Auditory-only and audiovisual paradigms should also be analyzed separately, as multisensory stimulation engages different neural systems and introduces photosensitivity-related safety considerations.

### Applications in attention-deficit hyperactivity disorder and autism spectrum disorder

ADHD and ASD are neurodevelopmental disorders, and frequently present as comorbidities (Antshel and Russo, 2019). Evidence suggests that ADHD symptomatology and associated cognitive deficits are commonly associated with aberrant neural oscillations (Deiber et al., 2020; Calderone et al., 2014; Woltering et al., 2012). Music may serve as an auxiliary strategy for the treatment of ADHD (Thaut et al., 2015; Vuilleumier and Trost, 2015; Luo and Zhang, 2025). Interventions based on music and rhythm have shown behavioral and cognitive effects in ADHD (Saville et al., 2025; Rickson, 2006; Jamey et al., 2025). However, the current evidence does not yet provide sufficient proof that these effects are achieved through the regulation of neural oscillations or entrainment. It is also worth noting that the above research has limitations, including a small sample size, a possible ceiling effect in the experimental task design, and a lack of controls.

ASD is an early-onset neurodevelopmental syndrome characterized by impairments in social communication, restricted interests, and sensory dysfunction (American Psychiatric Association, 2013). Altered neural oscillations have been observed across multiple EEG frequency bands in ASD (David et al., 2016). Additionally, studies have found that while children with ASD are capable of utilizing regular cues for temporal prediction, their neural entrainment to these cues is diminished (Beker et al., 2021). Other studies have found that children with ASD have reduced neural entrainment to rhythmic events (Beker and Molholm, 2023), as well as an impaired capacity to adapt to alterations in auditory rhythm (Vishne et al., 2021; Kasten et al., 2023). Music therapy has been shown to ameliorate certain ASD symptoms, such as improving language communication and social skills (Shi et al., 2024; Zhou et al., 2025; Ramirez-Melendez et al., 2022). However, these intervention measures, which incorporate music, social interaction, exercise, attention, and the therapist’s contact, do not typically directly manipulate or measure neural rhythm synchronization, and some of the results exhibit heterogeneity. They should therefore be interpreted as indirect clinical evidence. Practice guidance remains cautious about claims that music-based approaches reduce core ASD symptoms (Fuentes et al., 2021).

### Applications in depression

Depression is a major global public health issue. Evidence in depression spans physiology, acute stimulation, and small intervention studies. A cross-sectional study of first-episode major depression found lower right-hemisphere 40 Hz ASSR inter-trial phase coherence, while associations with symptom severity were not conventionally significant and no treatment was tested (Liu et al., 2023). An acute 33–48 Hz auditory frequency sweep in depression showed subgroup-dependent alpha-power differences and substantial inter-individual variation in peak response frequency, but did not assess clinical improvement or direct phase entrainment (Shi et al., 2025). A later study combined 40 Hz auditory pulses with adaptive audiovisual n-back training and reported greater improvement in MATRICS consensus cognitive battery (MCCB) scores than standard pharmacotherapy alone; because direct 40 Hz target engagement was not measured and the control did not match cognitive training or expectancy, the specific contribution of gamma stimulation remains uncertain (Wang et al., 2026). An open-label auditory-vibrotactile music intervention reported within-group improvements on the Montgomery-Åsberg Depression Rating Scale (MADRS) and resting-EEG changes, but lacked a control condition and did not measure stimulus-locked 40 Hz entrainment (Mosabbir et al., 2022). Thus, current findings in depression are best viewed as physiological or preliminary clinical signals rather than evidence of an established entrainment-mediated antidepressant effect.

### Safety

Safety assessment should be treated as a core translational endpoint rather than an incidental observation. Studies should report sound-pressure level at the ear, carrier and modulation frequencies, beat or event rate, session duration, cumulative exposure, device calibration, and hearing-protection procedures. Baseline and follow-up hearing status should be assessed, particularly in older adults and in protocols involving prolonged or repeated stimulation. Sensory hypersensitivity, tinnitus, vestibular symptoms, headache, migraine, discomfort, sleep disruption, and treatment-related anxiety should be systematically monitored. Audiovisual protocols also require screening and monitoring for photosensitivity and seizure risk.

Adherence, dropout, and reasons for discontinuation should be reported by group. Observed treatment-emergent adverse events must be distinguished from isolated case reports and theoretical risks. Studies should also specify whether adverse events were solicited using a structured instrument or recorded only when spontaneously reported. Tolerability reported in small pilot studies is generally acceptable but does not establish safety for longer exposure, higher intensity, vulnerable populations, or home use.

## Discussion

The literature on frequency bands supports a complementary, multiscale account of auditory processing. The correlation between different frequency bands is an effective organizing principle, but its functional specificity is limited. Frequency boundaries differ across studies, individual peak frequencies vary, and identical nominal frequencies can arise from different generators or analytic procedures. A central unresolved issue is whether a stimulus-locked oscillatory response reflects entrainment of endogenous activity or the repetition of evoked responses. ASSRs and neural tracking are valuable target-engagement measures, but stronger mechanistic claims require converging evidence. Reliability is also important: a measure that is unstable within individuals cannot support personalized stimulation or mediation analyses. Cross-frequency effects require parallel controls for non-sinusoidal waveforms, harmonics, filtering, and shared evoked transients.

The translational evidence is uneven. Animal models provide mechanistic evidence, and human studies show that frequency-targeted stimulation can elicit measurable neural responses. Clinical efficacy remains uncertain. Most studies are small, use heterogeneous devices and stimulation parameters, have short follow-up, and assess multiple outcomes. Sham conditions may be perceptually distinguishable, complicating expectancy control and participant blinding. Null findings, variable target engagement, selective effects on physiological rather than clinical endpoints, and potential publication bias should be reported alongside positive findings. Improvement after rhythm training or music therapy cannot be attributed to neural entrainment without demonstrating that the neural target changed and that this change mediated the clinical outcome.

In future research, emphasis should be placed on the causal chain, from stimulus parameters to neural target activation, to changes at the network level, and finally to behavioral or clinical changes. Three experiments are especially informative. First, adequately powered randomized trials should use credible sham or active controls that isolate the frequency-specific component from music, vibration, cognitive training, therapist contact, and expectancy. Second, fixed-frequency stimulation (including 40 Hz) should be compared with individualized frequencies using preregistered, within-person target-engagement thresholds and test–retest reliability. Third, mediation designs should determine whether the magnitude of neural target engagement predicts and statistically mediates clinically meaningful change. These studies should include blinded outcome assessment, standardized acoustic reporting, hearing and safety monitoring, durability follow-up, and publication of null findings. Multisite replication will be needed before auditory neural entrainment can be considered an established therapeutic strategy.

### Key concepts

*Auditory neural entrainment*: the externally driven alignment or adjustment of ongoing neural oscillatory activity to the temporal structure of an auditory stimulus.

*Stimulus-following response/ASSR*: a neural response that follows periodic stimulation; useful for assessing physiological target engagement but not sufficient on its own to demonstrate endogenous entrainment.

*Neural tracking*: correspondence between neural activity and time-varying stimulus features such as a speech envelope; it can arise through multiple mechanisms, including evoked responses and oscillatory alignment.

*Cross-frequency coupling*: statistical coordination between activity at different frequencies; apparent coupling must be tested against waveform, harmonic, filtering, and common-input artifacts.
